# Treatment patterns and healthcare resource utilization among patients with hereditary angioedema in the United States

**DOI:** 10.1186/s13023-018-0922-3

**Published:** 2018-10-12

**Authors:** Marc A Riedl, Aleena Banerji, Michael E Manning, Earl Burrell, Namita Joshi, Dipen Patel, Thomas Machnig, Ming-Hui Tai, Douglas J Watson

**Affiliations:** 1 Division of Rheumatology, Allergy & Immunology, US HAEA Angioedema Center, University of California, San Diego, 8899 University Center Lane, Suite 230, San Diego, CA 92122 USA; 2 Massachusetts General Hospital, Boston, MA USA; 3 Medical Research of Arizona, Allergy, Asthma & Immunology Associates, Scottsdale, USA; 4 CSL Behring, King of Prussia, PA USA; 5 Pharmerit International, Bethesda, MD USA; 6 CSL Behring, Marburg, Germany

**Keywords:** Central venous access device, Claims data, Healthcare resource utilization, Hereditary angioedema, Intravenous C1-inhibitor, Real-world, Treatment patterns

## Abstract

**Background:**

Real-world data on usage and associated outcomes with hereditary angioedema (HAE)-specific medications introduced to the United States (US) market since 2009 are very limited. The purpose of this retrospective study was to evaluate real-world treatment patterns of HAE-specific medications in the US and to assess their impact on healthcare resource utilization (HCRU). This analysis used IMS PharMetrics PlusTM database records (2006–2014) of patients with HAE, ≥1 insurance claim for an HAE-specific medication, and continuous insurance enrollment for ≥3 months following the first HAE prescription claim.

**Results:**

Of 631 total patients, 434 (68.8%) reported C1-INH(IV) use; 396 (62.8%) reported using ecallantide and/or icatibant. There were 306 episodes of prophylactic use of C1-INH(IV) (defined by continuous refills averaging ≥1500 IU/week for ≥13 weeks) in 155 patients; use of ≥1 on-demand rescue medication was implicated during 53% (163/306) of those episodes. Sixty-eight (20.2%) of 336 C1-INH(IV) users eligible for the HCRU analysis were hospitalized at least once, and 191 (56.8%) visited the emergency department (ED). Eighteen patients (5.4%) had a central venous access device (CVAD); of these, 5 (27.7%) required hospitalization and 14 (77.7%) had an ED visit. The adjusted relative risk of hospitalization and/or ED visits for patients with a CVAD was 2.6 (95% CI: 0.17, 39.23) compared to C1-INH(IV) users without a CVAD.

**Conclusions:**

Despite widespread availability of modern HAE medications in the US, we identified a subset of patients requiring long-term prophylaxis who continue to be burdened by frequent rescue medication usage and/or complications related to the use of CVADs for intravenous HAE medication.

## Background

Hereditary angioedema with C1 inhibitor deficiency (C1INH-HAE; hereafter “HAE”) is a rare disorder of genetic origin caused by C1 inhibitor deficiency or decreased functional activity [1]. Recent epidemiologic studies support HAE prevalence ranges from 1 in 60,000 to 1 in 100,0000 people, though misdiagnosis and delayed diagnosis of HAE are common [2, 3]. The characteristic features of HAE include intermittent and generally unpredictable attacks characterized by edematous swelling which can be very painful (abdominal attacks), disfiguring (peripheral swelling), or even fatal (laryngeal attacks). The disease can impart a considerable personal burden, significantly reducing quality of life (QoL), both during and between attacks; further, attacks lead to absenteeism from activities during attacks for both patients and their caregivers and decreased educational and career advancement [4–9].

General treatment goals for HAE include minimizing morbidity and preventing mortality, as well as maximizing QoL for patients living with this chronic, burdensome disease [1]. These goals can be accomplished by effective “on-demand” treatment of attacks, as well as routine prophylaxis of attacks, if warranted. Prior to 2008, medications for the acute treatment of HAE in the United States (US) were limited to supportive treatment. There are now four US Food and Drug Administration (FDA)-approved on demand treatments for HAE attacks: intravenous (IV) plasma-derived C1-INH [C1-INH(IV) Berinert®, CSL Behring, Marburg, Germany]; subcutaneous (SC) icatibant (Firazyr®, Shire, Lexington, MA); SC ecallantide (Kalbitor®, Shire), and IV recombinant human C1-INH (Ruconest®, Pharming Healthcare, Inc., Berkeley Heights, NJ).

Prophylaxis options remain more limited, and historically included oral attenuated androgens and antifibrinolytic agents (eg, tranexamic acid). Long-term use of oral androgens, while convenient and inexpensive, are associated with a number of health risks, toxicities, and adverse side effects. Thus, they are generally not preferred for long-term prophylaxis, and are particularly unsuitable for young patients and women, especially during pregnancy or breast-feeding [1, 10–12]. Antifibrinolytics are not recommended because of their lower efficacy relative to other prophylactic options [1, 13]. Newer disease-specific options for HAE prophylaxis include plasma-derived C1-INH(IV) (Cinryze®, Shire ViroPharma, Lexington, MA), FDA-approved in 2008, a SC formulation of C1-INH (C1-INH[SC]; HAEGARDA®, CSL Behring, Marburg, Germany) approved by the FDA in June 2017, and a SC monoclonal antibody (lanadelumab; TAKHZYRO™, Shire, Lexington, MA) FDA-approved in August, 2018. Routine prophylaxis with C1-INH(IV) reduces the median and mean attack frequency by half [14], while C1-INH(SC) at the approved dose of 60 IU/kg was shown to reduce the median (mean) attack frequency by 95% (84%) [15].

The introduction of HAE-specific therapies for both on-demand and prophylaxis treatment represented major advancements in HAE disease management, and self-administration has been embraced as a safe and feasible practice. Despite these new treatments which can effectively alleviate the disease burden for many patients, IV medication use may pose certain challenges, including difficulty gaining and/or maintaining venous access, or logistical issues pertaining to proper infusion procedure; some patients are simply reluctant to self-administer IV medication because of physical or psychological barriers [16, 17]. The extent to which these factors influence outcomes are currently poorly understood.

Real-world data are particularly useful for evaluating medication usage patterns and can help to quantify certain relevant outcomes. While long-term studies, observational cohorts, and registry data have been published for individual HAE products [18–22], there are limited real-world data on general usage patterns encompassing all available disease-specific HAE medications in the US population, including concomitant medication usage patterns. Most notably, no studies have specifically evaluated outcomes associated with the use of IV versus non-IV medications.

The purpose of this retrospective study was to evaluate real-world treatment patterns of HAE-specific medications in the US and to assess their impact on healthcare resource utilization (HCRU).

## Methods

### Data source

This non-interventional, retrospective cohort study was conducted using de-identified data from the IMS PharMetrics Plus™ database (formerly IMS LifeLink™ Health Plan Claims Database) for the period January 1, 2006 to December 31, 2014. The PharMetrics Plus database captures paid claims to health providers for over 80 insurance and managed care plans throughout the US including inpatient and outpatient care. The data available include health plan enrollment information, demographic characteristics, diagnosis, diagnostic procedures, lab tests, and prescription drug use. The data are representative of the US national commercially insured population in terms of age and sex, including adjudicated claims for more than 47 million unique enrollees per year. The PharMetrics Plus database is fully compliant with the Health Insurance Portability and Accountability Act of 1996 (HIPAA) privacy regulations. As all patient-level data are HIPAA-compliant and certified anonymous, Institutional Review Board approval and patient informed consent were not required for this study. In order to comply with HIPAA, the databases were de-identified to preserve patient anonymity and confidentially.

### Study cohort

**Fig. 1:**
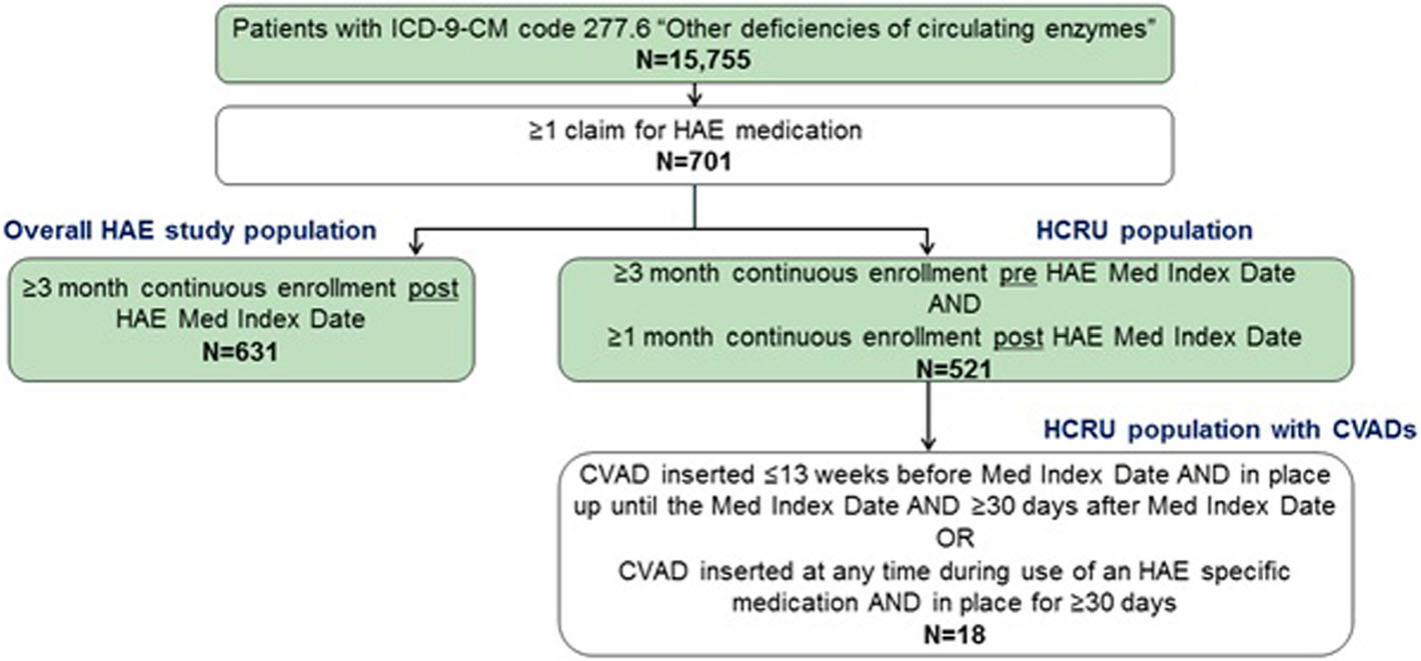
Study design and patient inclusion criteria. CVAD, central venous access device; HAE, hereditary angioedema; HCRU, health care resource utilization; ICD-9-CM, International Classification of Diseases, 9th Revision, Clinical Modification Med Index Date, first recorded HAE prescription claim during the study period

Criteria for inclusion in the overall HAE study cohort included a recorded diagnosis of HAE (InternationaI Classification of Diseases, 9th Revision, Clinical Modification [ICD-9-CM] code 277.6, “other deficiencies of circulating enzymes”) and ≥ 1 prescription claim(s) for an HAE-specific medication [C1-INH(IV)/Cinryze®), C1-INH(IV)/Berinert®, icatibant, ecallantide during the study period (Fig. 1). Recombinant C1-INH(IV)/Ruconest was not included in the analyses because of its late approval (July 2014) relative to the study period, and C1-INH(SC) was not yet available. Since the focus of this research was the use of the more recently introduced HAE-pecific medications, patients using oral androgens or tranexamic acid as their only medication(s) for HAE were not included. Continuous health plan enrollment for ≥3 months after the first recorded HAE prescription claim during the study period (“medication index date”) was also required for inclusion in the overall cohort. In addition to the overall cohort, there was a HCRU analysis cohort, which was a subset of the overall cohort. Additional eligibility criteria for the HCRU cohort included ≥3 months of continuous health plan enrollment prior to the HAE medication index date and ≥1 month continuous enrollment after the HAE medication index date. The HCRU cohort was further sub-divided into two cohorts: a HCRU cohort with and without any central venous access device (CVAD) use (identified as SC port intended for long-term infusion therapy using current procedural terminology [CPT®] codes).

The HCRU-CVAD cohort included patients with either of the following claim patterns: a claim for a CVAD inserted ≤13 weeks before the HAE medication index date and in place until, and for at least 30 days after the HAE medication index date; or, a claim for a CVAD inserted at any time during the use of an HAE-specific medication and in place for 30 days or longer.

### C1-INH(IV) users and HAE rescue treatment assessment

For this analysis, prescription data were evaluated based on the general assumption that filled prescriptions equated to medication usage by the patients. For on-demand medications, this assumption may lead to higher estimates of actual usage, since on-demand medication is also standby medication, not all of which is necessarily consumed by the patients. Also, due to the inability to definitively categorize intended medical use of C1INH(IV) (on-demand vs prophylactic use), routine prophylactic C1-INH(IV) use was presumed based on minimum dose threshold and refill duration patterns. Prophylactic C1-INH(IV) use was defined by convention as continuous refills of C1-INH(IV) averaging ≥1500 IU/week for ≥13 consecutive weeks. Based on C1-INH(IV) product labeling, 1000 IU every 3 or 4 days is the recommended dosing for prophylaxis [23]; thus, ongoing usage at the lower end of this range (every 4 days) would average 1750 units per week. For this analysis, the designated minimum threshold of 1500 IU per week, combined with the required duration of this average refill activity over a period of at least 13 weeks, was determined to be reasonable for identifying ongoing, assumed prophylaxis use at or above the minimum recommended dosage, as well as ongoing usage slightly below that minimum to allow for the likelihood of real-world, individualized prophylaxis dosing regimens. The period from the start to end of such continuous refill activity qualified as an “episode” of assumed routine prophylaxis. Each episode fulfilling these criteria was assumed to represent a period of prophylactic C1-INH(IV) treatment for data analysis purposes.

On-demand C1-INH(IV) use was defined as C1-INH(IV) refilled at an average of <1500 IU/week for ≥13 weeks; this treatment episode was presumed to be on-demand use. Based on these definitions, one subject could have more than one episode of prophylactic C1-INH(IV) use and more than one period of on-demand use over the course of the study.

During periods of prophylactic C1-INH(IV) use, “rescue treatment” (on-demand use of HAE medication) was defined as any use of the following: ecallantide, icatibant, or a different C1-INH(IV) product than that identified as being used for prophylaxis.

### Health care resource utilization analysis

HCRU outcomes of interest included hospitalizations, emergency department (ED) visits, and implantation of CVADs. CVAD use was deemed to be related to the use of IV HAE medication if it was inserted prior to, or at any time during the use of an HAE medication, and in place for at least 30 days.

HCRU was classified as all-cause, CVAD-related, and/or HAE-related (using primary/secondary diagnosis of HAE identified using ICD-9-CM codes). CVAD-related complications were identified using CPT codes for CVAD insertion, repair, partial replacement, complete replacement, and removal. All-cause HCRU was compared between patients with and without a CVAD.

In order to account for disease severity and comorbid conditions in the analysis prior to initiation of HAE treatment, the Charlson comorbidity index (CCI) score was computed for each patient using the Deyo adaptation [24]. The CCI provides a means of evaluating comorbidity impact, specifically as a predictor of one-year mortality risk, taking into account the number and seriousness of comorbid conditions [25]. Comorbidities were flagged using ICD-9-CM codes and predetermined weights were applied to each comorbidity and summed to create an aggregate CCI summary score.

To assess the baseline comorbidity burden, the CCI score was computed for each patient in the period 6 months prior to the HAE medication index date.

### Statistical analysis

A generalized linear model was used to examine the risk of all-cause and HAE/CVAD-related HCRU during the 12-month follow up period after the HAE medication index date. The association between HCRU (office visits, hospitalizations, and ED visits) and the use of a CVAD (vs none) was examined during the study period adjusting for age, geographic region, initial treatment course, payer type, insurance type, CCI, and pharmacy benefit. Some of the categories were collapsed for geographic region, payer, and insurance type variables due to small sample size. Propensity score matching was employed to balance the baseline differences in demographic and clinical characteristics between CVAD and non-CVAD users. Propensity scores (the estimated probability of initiating/using a CVAD) were calculated using a multivariable logistic regression model for all patients in the HCRU cohort. A matching algorithm was then used to match (1:1) patients with a CVAD to those without a CVAD.

Statistical tests of significance were two-sided, with a *p*-value <0.05 considered significant. All statistical analyses were performed using SAS 9.4 (SAS Institute Inc., Cary, NC).

## Results

### Study cohort

A total of 631 patients with HAE (71% female, mean age 38.3 years) were identified as meeting the inclusion criteria of this study (Table 1). A majority (89.5%) of patients were of working age (17 to <65 years), which was not unexpected given the commercial insurance nature of the PharMetrics Plus database. The mean CCI score in the study population was low at 0.35 (range of possible scores, 0 [no comorbidity burden] to 25).

**Table 1:** Baseline characteristics of study cohort

	All HAE patients (*N* = 631)
Sex, n (%)	
Female	448 (71.0)
Male	183 (29.0)
Age (years), mean (SD)	38.3 (15.3)
Age Distribution, *n* (%)	
<12 years (%)	17 (2.7)
12 to <17 years (%)	27 (4.3)
17 to <65 years (%)	565 (89.5)
≥65 years (%)	22 (3.5)
Geographic region of the US, *n* (%)	
East	171 (27.1)
Midwest	167 (26.5)
South	249 (39.5)
West	44 (7.0)
Payer Type, *n* (%)	
Commercial	452 (71.6)
Self-insured	160 (25.4)
Medicaid	13 (2.1)
Medicare Risk	2 (0.3)
Other^a^	3 (0.5)
Missing/unknown	1 (0.2)
Charlson Comorbidity Index, mean (SD)	0.35 (0.78)

*HAE* hereditary angioedema, *SD* standard deviation

^a^Other included 1 patient (0.2%) each with the following: Medicare Cost (supplemental), State Children’s Health Insurance Program, Rx Only

### HAE medication use

Table 2 summarizes HAE medication usage patterns over the course of the study period. Any use of C1-INH(IV) was observed in 68.8% (*n* = 434) of patients, about half of whom also received icatibant or ecallantide. About one-third (31.2%; *n* = 197) of patients exclusively had claims for SC HAE medication(s) (icatibant or ecallantide) without concomitant use of C1-INH(IV). Exclusive use of C1-INH(IV) was observed in 37.2% of patients, and the remaining 31.5% of patients had claims for both SC and IV medications.

**Table 2:** HAE-specific medications used during study period (2006–2014) in 631 patients with HAE in the US^a^

HAE Medications	*N* (%) of Patients (*N* = 631)
Icatibant and/or ecallantide only	197 (31.2)
Any use C1-INH(IV)	434 (68.8)
C1-INH(IV)/Cinrzye	110 (17.4)
C1-INH(IV)/Cinryze + icatibant/ecallantide	97 (15.4)
C1-INH(IV)/Berinert	87 (13.8)
C1-INH(IV)/Berinert + icatibant/ecallantide	53 (8.4)
C1-INH(IV)/Cinryze + C1-INH(IV)/Berinert + icatibant/ ecallantide	49 (7.8)
C1-INH(IV)/Cinryze + C1-INH(IV)/Berinert	38 (6.0)

*HAE* hereditary angioedema

^a^All HAE-specific medications recorded during study period

There were 306 episodes of prophylactic use of C1-INH(IV) in 155 (24.6%) patients with a mean duration of 339 (median, 245 days; range, 91–1891 days). Use of at least one rescue medication was observed during 53% (163/306) of prophylactic episodes. The most common rescue medications were icatibant (25% of episodes) and a C1-INH(IV) product other than that being used for prophylaxis (25% of episodes).

### Central venous access device use and complications

A total of 521 patients contributed to the HCRU analysis. Among 336 C1-INH(IV) users in the HCRU analysis, 18 (5.4%) were identified as having a CVAD (all SC ports) deemed related to their use of C1-INH(IV). Ten (55.6%) of the 18 patients with a CVAD had at least 1 major complication leading to CVAD replacement or repair (Table 3).

**Table 3:** Prevalence of CVAD-related complications^a^

CVAD-related complication	Patients with a CVAD *N* = 18
Any complication, *n* (%)	10 (55.6)
Specific complications, *n* (%)^a^	
Mechanical complication	7 (38.9)
Removal of pericatheter and insertion of replacement catheter	7 (38.9)
Other and unspecified infection due to central venous catheter	2 (11.1)
Local infection due to central venous catheter	1 (5.6)

*CVAD* central venous access device

^a^A given patient may have had more than one CVAD-related complication

### Hospitalization and emergency department visits

Overall, 477 of the 521 patients (91.6%) in the HCRU analysis had one or more doctor’s office visits, 79 (15.2%) experienced one or more hospitalizations, and 271 (52.0%) had one or more ED visits during the study period. Users of C1-INH(IV) (*n* = 336; 64.5%) had higher crude rates of hospitalization (20% vs 6%) and ED visits (57% vs 43%) compared with patients not using an HAE medication requiring IV access (Table 4).

**Table 4:** Crude (unadjusted) rates of healthcare resource utilization (HCRU) stratified by C1-INH(IV) use/non-use and by CVAD use/non-use

	HCRU *n* (%) of patients with any HCRU^a^	
	Hospitalization	ED Visit
Overall (*n* = 521^b^)	79 (15.2)	271 (52.0)
By C1-INH(IV) use		
C1-INH(IV) users (*n* = 336)	68 (20.2)	191 (56.8)
Non-C1-INH(IV) users (*n* = 185)	11 (5.9)	80 (43.2)
By CVAD use		
CVAD users (*n* = 18)	5 (27.8)	14 (77.8)
Non-CVAD users (*n* = 503)	74 (14.7)	257 (51.1)

*CVAD* central venous access device, *ED* emergency department, *HCRU* healthcare resource utilization

^a^During study period; percentages reflect row %

^b^Total HCRU analysis population

Among the 18 CVAD users, 5 (27.8%) required hospitalization during the study period compared to 74 (15%) non-CVAD patients (unadjusted data). Fourteen (77.8%) CVAD patients visited an ED during the study period, compared to 257 (51.1%) non-CVAD patients (Table 4). In the propensity score-matched analysis in which 15 of the 18 CVAD users were matched 1:1 to non-CVAD user controls, the adjusted relative risk of hospitalization or ED visit was still higher in CVAD users compared to non-CVAD users (relative risk 2.6; 95% CI 0.17, 39.23).

## Discussion

This study of 631 unique patients is the largest study of its kind to evaluate real-world treatment patterns and outcomes in a US cohort of patients with HAE. During the study time window (2006–2014), C1-INH(IV), either as on-demand or prophylactic treatment, was the most frequently used medication for management of patients with HAE in the US. Slightly more than two-thirds of patients had claims for C1-INH(IV) during the 9-year study period. Our data suggest that C1-INH(IV) was used for routine HAE prophylaxis by at least 25% of patients, with the caveat that due to the nature of the prescription data analyzed, intended use (prophylactic vs on-demand) could only be assumed. As a surrogate means of assigning intention of use, we devised parameters for defining prophylactic use based on C1-INH(IV) refill activity. In more than half of episodes that were identified as prophylactic episodes, claims for ongoing prescriptions of HAE on-demand medications were observed. This is likely to reflect the implementation of current US HAE guidelines stating patients on a prophylactic treatment regimen must also have access to effective on-demand treatment for acute attacks [1]. These observations may also corroborate findings from a recent HAE patient survey which included 47 users of C1-INH(IV) for routine HAE prophylaxis who reported breakthrough attacks with a frequency of at least once per month [16].

The use of CVADs such as subcutaneous ports is strongly discouraged by the US Hereditary Angioedema Association Medical Advisory Board unless deemed as a last resort [1]. It is well-known that CVADs are associated with a number of medical risks including infections and thrombotic complications [26–29]. Up until recently, only anecdotal reports were available describing complications in HAE patients with ports, including thrombosis and systemic fungal infection [30]. Based on our study we estimated the prevalence of port use in the US HAE population during the study period who used regular C1-INH(IV) infusions to be around 5% and that more than half of those patients with a CVAD experienced at least one major complication leading to CVAD replacement or repair. A similarly high risk of CVAD complications was reported in a recent patient survey which included HAE patients with ports and weekly intravenous C1-INH infusions [16]. Our data also revealed higher levels of healthcare utilization (eg, hospitalizations, ED visits) in the subgroup of patients who were port users. However, an important limitation of interpreting these findings is that the data could not determine causality between CVAD use and reasons for the higher rates of healthcare visits. It is certainly possible that patients with CVAD had more severe disease and/or had other underlying risk factors that predisposed them to more frequent hospitalization and/or emergency care, and issues other than port use likely contributed to this finding.

There are several additional limitations that need to be considered when interpreting the findings presented herein. Most notably, claims data do not provide a definitive means of identifying intent of medication usage. Therefore, while medication claims were interpreted as usage and a surrogate marker for breakthrough attacks for the purposes of this analysis, it is understood that prescription claims may not always accurately indicate clinical medication usage or HAE attack patterns. For analysis purposes, claims for on-demand medications that occurred during periods of prophylaxis were assumed to indicate breakthrough attacks, despite the possibility that some of these claims may have been for medications to keep on hand in case of an attack, thus exaggerating the interpreted prevalence of breakthrough attacks. Conversely, it is likely that many patients using C1-INH(IV) for routine prophylaxis may have used the same product to treat breakthrough attacks; this “rescue” usage could not have been differentiated from the prophylaxis refill activity of the same product and as a result, the number of breakthrough attacks may have been under-estimated in such cases. It is also possible that some patients requiring large doses of C1-INH(IV) as frequent on-demand treatment could have been incorrectly categorized as using regular prophylaxis based on the surrogate threshold of ≥1500 IU/week for ≥13 weeks. In addition, surrogate definitions based on sustained refill patterns exceeding a defined threshold were used to categorize prophylactic use of C1-INH(IV). Finally, since the claims database used in this study included only commercially-insured patients in the US, individuals that are uninsured or covered under government health plans, including the elderly, are under-represented and thus generalizability of the findings to the overall population of the US, as well as to other countries, is limited.

## Conclusions

This analysis of a large real-world claims database suggests that, despite the introduction of multiple new HAE-specific medications in the US since 2008, a subset of patients with HAE requiring long-term prophylaxis continue to experience considerable disease and treatment burden. Specific treatment burdens suggested by these data include sub-optimal attack prevention efficacy and the need for central venous access in some US patients, along with corresponding higher consumption of hospital and emergency healthcare services.

## Abbreviations

C1-INH-HAE: Hereditary Angioedema with C1 inhibitor Deficiency
CCI: Charlson Comorbidity Index
CVAD: Central Venous Access Device
ED: Emergency Department
FDA: US Food and Drug Administration
HAE: Hereditary Angeiodema
HCRU: Healthcare Resource Utilization
ICD-9-CM: International Classification of Diseases, 9th Revision, Clinical Modification
IV: Intravenous
QoL: Quality of Life
SC: Subcutaneous
US: United States
